# Low Doses of Mycotoxin Mixtures below EU Regulatory Limits Can Negatively Affect the Performance of Broiler Chickens: A Longitudinal Study

**DOI:** 10.3390/toxins12070433

**Published:** 2020-07-01

**Authors:** Oluwatobi Kolawole, Abigail Graham, Caroline Donaldson, Bronagh Owens, Wilfred A. Abia, Julie Meneely, Michael J. Alcorn, Lisa Connolly, Christopher T. Elliott

**Affiliations:** 1 Institute for Global Food Security, School of Biological Sciences, Queens University, Belfast BT9 5DL, UK; oluwatobi.kolawole@qub.ac.uk (O.K.); W.Abia@qub.ac.uk (W.A.A.); j.p.meneely@qub.ac.uk (J.M.); l.connolly@qub.ac.uk (L.C.); 2 Devenish Nutrition Limited, Lagan House, 19 Clarendon Road, Belfast BT1 3BG, UK; abigail.graham@devenish.com (A.G.); caroline.donaldson@devenish.com (C.D.); bronagh.owens@devenishnutrition.com (B.O.); mike.alcorn@devenish.com (M.J.A.)

**Keywords:** multi-mycotoxins, animal feed, animal health, chronic exposure, feed efficiency, LC-MS/MS, statistical analysis

## Abstract

Several studies have reported a wide range of severe health effects as well as clinical signs, when livestock animals are exposed to high concentration of mycotoxins. However, little is known regarding health effects of mycotoxins at low levels. Thus, a long-term feeding trial (between May 2017 and December 2019) was used to evaluate the effect of low doses of mycotoxin mixtures on performance of broiler chickens fed a naturally contaminated diet. In total, 18 successive broiler performance trials were carried out during the study period, with approximately 2200 one-day-old Ross-308 chicks used for each trial. Feed samples given to birds were collected at the beginning of each trial and analysed for multi-mycotoxins using a validated LC-MS/MS method. Furthermore, parameters including feed intake, body weight and feed efficiency were recorded on a weekly basis. In total, 24 mycotoxins were detected in samples analysed with deoxynivalenol (DON), zearalenone (ZEN), fumonisins (FBs), apicidin, enniatins (ENNs), emodin and beauvericin (BEV), the most prevalent mycotoxins. Furthermore, significantly higher levels (however below EU guidance values) of DON, ZEN, FBs, BEV, ENNs and diacetoxyscirpenol (DAS) were detected in 6 of the 18 performance trials. A strong positive relationship was observed between broilers feed efficiency and DON (R^2^ = 0.85), FBs (R^2^ = 0.53), DAS (R^2^ = 0.86), ZEN (R^2^ = 0.92), ENNs (R^2^ = 0.60) and BEV (R^2^ = 0.73). Moreover, a three-way interaction regression model revealed that mixtures of ZEN, DON and FBs (*p* = 0.01, R^2^ = 0.84) and ZEN, DON and DAS (*p* = 0.001, R^2^ = 0.91) had a statistically significant interaction effect on the birds’ feed efficiency. As farm animals are often exposed to low doses of mycotoxin mixtures (especially fusarium mycotoxins), a cumulative risk assessment in terms of measuring and mitigating against the economic, welfare and health impacts is needed for this group of compounds.

## 1. Introduction

Animal feed plays a significant role in global food industry as it is one of the most important components for sustainable production of safe and affordable animal proteins [1,2]. However, feed is routinely subjected to a wide range of natural and synthetic environmental contaminants, which may pose serious risk to animal and human health [3]. Among the vast numbers of harmful contaminants in feed, mycotoxins are an important natural feed contaminant with potential to induce negative effects on animal health, welfare and productivity [1]. Mycotoxins are toxic secondary metabolites produced by some specific species of filamentous fungi, mainly belonging to genera of *Aspergillus, Alternaria, Fusarium, Clavicep* and *Penicillium* [4]. These fungi infect crops such as cereals, vegetables and fruits pre- and post-harvest, thereby causing severe reduction in the yield and damage the quality of the crop [1,4]. Currently, more than 400 mycotoxins have been identified with increasing numbers, due to the impact of climate change and agricultural practices [5,6].

Farm animals are exposed to mycotoxins mainly through ingestion of contaminated feed [7]. Several reports are available on adverse health effects of mycotoxin in livestock animals [7,8]; the severity and symptoms observed were dependent on the type of mycotoxin, level of exposure, duration, sex, age, animal species and interaction with other contaminants in feed [7,8]. Furthermore, long-term exposure of farm animals to mycotoxin-contaminated feed may lead to deposition of mycotoxin residues in animal tissues and organs as well as carry-over to animal products such as meat, milk and egg. This may result in indirect human intake of mycotoxins [8,9].

To mitigate the impact of mycotoxins on human and animal health as well as the economy, regulations have been established in many parts of the world, mainly for six mycotoxins which frequently contaminate agricultural commodities and with potential to induce severe toxic effects in farm animal [10]. Compared to the rest of the world, the European Union (EU) has the strictest mycotoxins regulations. A maximum permitted level of 0.02 mg/kg for aflatoxin B1 (AFB1), and a guidance or recommended values of 5, 0.25, 0.25, 0.1 and 20 mg/kg were set for deoxynivalenol (DON), zearalenone (ZEN), T-2, ochratoxin A (OTA) and fumonisins (FB1+FB2), respectively, in poultry feed [11]. However, other frequently occurring mycotoxins commonly known as emerging mycotoxins including enniatins, beauvericin and moniliformin have not been regulated. They are considered to be of less importance in terms of levels at which they naturally occur in food and feed as well as their toxic effects in human and animal [12,13]. In addition to the emerging mycotoxins, another group of mycotoxins that have recently emerged as an important co-contaminant in feedstuffs are plant-derived conjugates called masked mycotoxins. These mycotoxins are biologically modified phase II metabolites formed by plant defence mechanisms through glycosylation catalysed by uridine diphosphate-glucosyltransferases [14,15]. The most frequently detected in animal feed include deoxynivalenol-3-glucoside, nivalenol-3-glucoside, zearalenol-14-glucoside, α-zearalenol-14-glucoside, β-zearalenol-14-glucoside T-2-toxin-3-glucoside and HT-2-toxin-3-glucoside [14,15]. A few studies have shown that these compounds can be converted in the gastrointestinal tract (GIT) from the conjugated to the free form, leading to increased toxicity [16,17]. Regulations are yet to be set for this group of mycotoxins, due to insufficient occurrence and toxicological data [15].

As reported in several national, regional and worldwide mycotoxin surveys, feed and feedstuffs are frequently contaminated with multiple mycotoxins (both regulated and non-regulated) at higher levels when compared to foods [5,18,19,20]. This is because complete feeds are made from a blend of materials including cereals and soybean, which are more susceptible to pre- and post-harvest fungal infection. In addition, two or more fungi can simultaneously infect crops and some fungal species can produce more than one mycotoxin [16]. In terms of health effects due to multi-mycotoxin exposures, several studies have suggested an interaction which may lead to additive, antagonistic and synergistic effects [21]. Exposure of poultry to either single or multiple mycotoxins (mainly AFB1, DON, ZEN, FB1, OTA and T-2) led to various symptoms, with more pronounced adverse health effects in birds exposed to multiple toxins compared to single toxin exposure. Exposure of broilers to diet contaminated with 6.5 mg/kg DON, 0.47 mg/kg 15-acetyl-DON and 0.73 mg/kg ZEN showed significantly reduced feed intake and lowered CD4+ and CD8+ cells at Days 3 and 14 compared to birds fed uncontaminated diet [22]. Similarly, a broiler diet containing up to 0.4 mg/kg AFB1, 0.2 mg/kg OTA and 0.3 mg/kg DON caused reduced feed intake and feed efficiency as well as antibody titre against Newcastle disease and infectious bursal diseases [23]. Filho et al. [24] also observed a decrease in feed intake (35%) and body weight (50%) in broiler chicken exposed to 2 mg/kg AFB1 and 100 mg/kg FB1 [24]. A diet containing a mixture of T-2 (0.2–2.2 mg/kg) and DON (4.9–24.9 mg/kg) induced oxidative stress in the liver of chicken and decreased lipid peroxidation [25]. In broiler production, mycotoxin-contaminated feed may cause a significant economic loss for poultry producers, due to deleterious effects on animal health and performance as well as increased production costs.

Across Northern Ireland, the levels of mycotoxin in feed materials such as wheat and soybean are generally low due to the intensive testing and monitoring programme provided by the food fortress scheme initiative [26]. Consequently, finished feeds in the supply chain are compliant with EU regulatory limits for mycotoxins in feed. Since most of the available studies on broiler chicken performance following mycotoxin exposure are focused on only regulated mycotoxins at levels far above EU limits or not relative to all field conditions, very little is known regarding the effect of low to moderate levels of mycotoxin mixtures on broiler production. Thus, the objectives of this study were to carry out long-term performance feeding trials, to determine the degree of chicken exposure to multiple mycotoxins and to compare the impact of mycotoxin levels found in feed samples of each performance trial on zootechnical parameters of broiler chicken. Furthermore, a multi-mycotoxin LC-MS/MS method was developed and validated for an accurate screening and monitoring of 56 mycotoxins in broiler feed samples.

## 2. Results

A QuEChERS-based LC-MS/MS method was developed and validated for the extraction and analysis of multiple mycotoxins in poultry feed samples. Overall, performance characteristics (linearity, sensitivity, recovery, precision and specificity) of the method met criteria set by the European Commission Regulation No. 657/2002 and 401/2006 [27,28] (Tables S1 and S2). The validated method was then applied for the analysis of multiple mycotoxins in 78 poultry feed samples. In total, 24 fungal metabolites were detected in the feed samples analysed (Table 1). The most frequently occurring mycotoxins were DON, ZEN, FBs (FB1 + FB2 + FB3), beauvericin (BEV), apicidin (APD) and enniatins (ENN A, A1, B and B1). FBs and ENNs were prevalent in 100% of the samples, while DON, BEV, ZEN and APD contaminated 98%, 95%, 91% and 85% of the samples, respectively. In terms of regulated mycotoxins, only DON, ZEN and FBs were found to contaminate the feed samples at levels well below the EU guidance values for these toxins in poultry feed (Figure S1). The maximum concentrations of DON, ZEN and FBs found in the samples were 2.62, 0.24 and 3.58 mg/kg, respectively. Regarding non-regulated mycotoxins, ENNs, BEV and APD were found in all samples analysed at maximum levels of 2.65, 0.47 and 0.064 mg/kg, respectively. ENNB was the most abundant and frequently occurring ENNs, followed by ENNB1, ENNA and ENNA1. Furthermore, tentoxin (TET), meleagrin (MEG), alternariol, citrinin, moniliformin (MON) and patulin were found in up to 65% of feed samples, at levels ranging from limit of quantification to 0.39 mg/kg. Other mycotoxins including diacetoxyscirpenol (DAS), mycophenolic acid, aurofurasin (AUR), equisetin (EQS), patulin (PAT), emodin (EMD) and roquefortine C (ROC) were detected in about 55–60 (70–83%) of samples analysed, at levels ranging from 0.002 to 0.89 mg/kg. Deoxynivalenol-3-glucoside and 3-Acetyl-deoxynivalenol were the only modified mycotoxins detected in more than 50% of the samples at maximum levels of 0.09 and 0.15 mg/kg, respectively. Up to 90% of the samples were contaminated with more than 10 mycotoxins, and about 75% of the samples contained at least 20 mycotoxins. The most frequent mixture was DON, ZEN, FBs, ENNs, BEV, TET, APD, EMD and AUR.

### 2.1. Animal Performance

**Figure 1:**
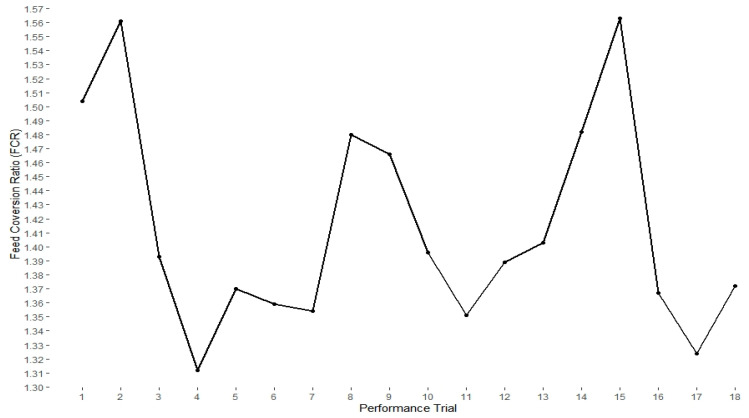
Feed conversion ratio (FCR) of broiler chickens fed naturally contaminated diet. FCR of broilers in each performance trial was calculated as a ratio of total feed consumed to live body weight of chicken at the end of the trial.

The average feed intake and average body weight recorded for birds across performance trials are shown in Table 2. A significant positive correlation (*p* < 0.05) between feed intake and body weight was observed on Days 7, 14 and 21. However, during Days 22–28, no significant relationship was observed between feed intake and body weight of broiler chickens (Table 2). The FCR of broilers in each trial was calculated as a ratio of total feed consumed to live body weight at the end of the trial. A significantly higher FCR was recorded for birds in performance Trials 1, 2, 8, 9, 14 and 15 (*p* < 0.05), when compared with birds in other performance trials (Figure 1). Regarding mortality, the highest mortality was recorded for birds in Trial 1 (6%), the lowest for birds in Trial 15 (1%), while other trials had mortality rate between 2% and 4%. No relationship was found between mortality and broilers exposure to mycotoxins.

### 2.2. Impact of Multiple Mycotoxin on Broilers Performance

**Figure 2:**
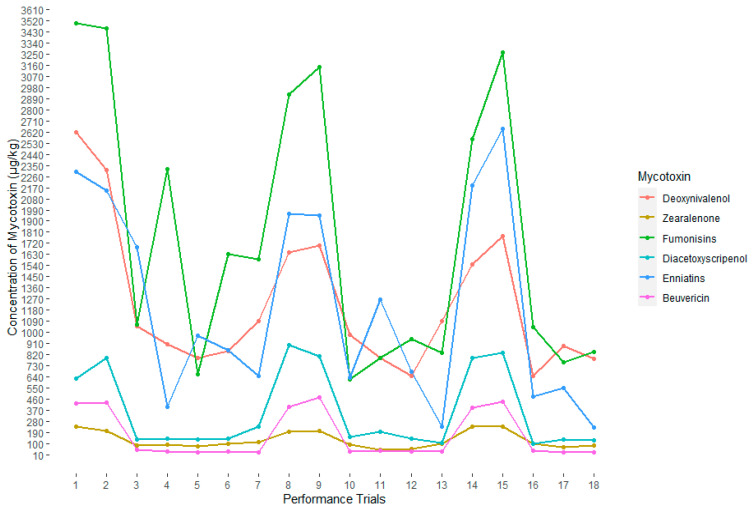
Mean concentrations of deoxynivalenol, zearalenone, fumonisins, diacetoxyscirpenol, enniatins and beauvericin in broiler feed samples collected from May 2017 to December 2019. In total, 18 successive performance trials were carried out during the study period.

**Figure 3:**
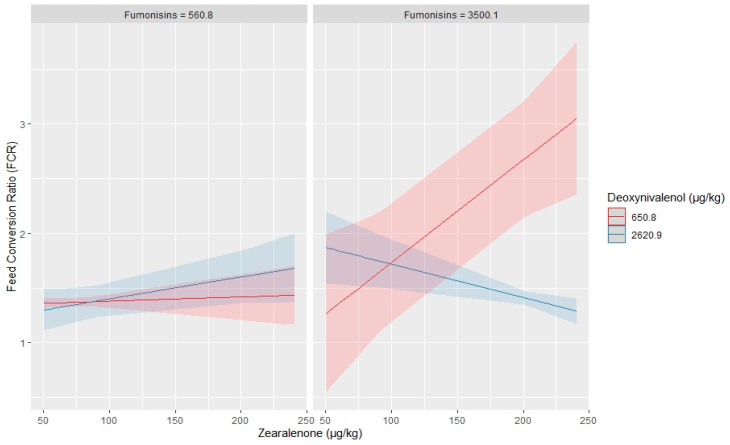
A three-way interaction plot with predicted values of broilers feed efficiency (FCR) based on level of occurrence and interaction effect among mixtures of zearalenone, deoxynivalenol and fumonisins. The shaded area around the fitted effects represent the 95% confidence interval. Deoxynivalenol and diacetoxyscirpenol (moderator variables) were held constant at minimum and maximum values.

**Figure 4:**
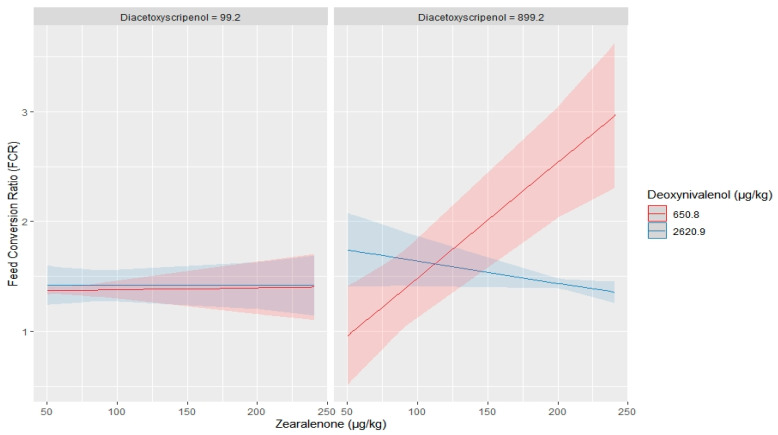
A three-way interaction plot with predicted values of broilers feed efficiency (FCR) based on level of occurrence and interaction effect among mixtures of (zearalenone, deoxynivalenol and diacetoxyscirpenol). The shaded area around the fitted effects represent the 95% confidence interval. Deoxynivalenol and diacetoxyscirpenol (moderator variables) were held constant at minimum and maximum values.

Mean mycotoxin levels in feed samples given to birds in each performance trial were analysed using a two-way analysis of variance, with post-hoc tests. The results show that DON, ZEN, FBs, ENNs, BEV and DAS were the only mycotoxins with significantly higher levels in feed samples given to birds in Trials 1, 2, 8, 9, 14 and 15 (*p* < 0.05) (Figure 2). A strong positive relationship (*p* < 0.05) was observed between FCR and DON (R^2^ = 0.85), FBs (R^2^ = 0.53), DAS (R^2^ = 0.86), ZEN (R^2^ = 0.92), ENNs (R^2^ = 0.60) and BEV (R^2^ = 0.73). Furthermore, a multiple linear regression model was used to explore the relationship between mycotoxin interaction and effect on broilers FCR. Three-way interaction regression models were fitted, with FCR as dependent variable and the six toxins as independent variables. The order of interaction term was based on the toxicological relevance or sensitivity of broilers to mycotoxins (i.e., ZEN > DON > FBs > ENNs > BEV > DAS), thus ten possible three-way interaction terms were evaluated (Table S3). Only the interactions among ZEN, DON and FBs (*p* = 0.01, R^2^ = 0.84) as well as ZEN, DON and DAS (*p* = 0.001, R^2^ = 0.91) had a significant effect on broilers FCR. None of the other interactions were significant. Predicted values of FCR based on the level of occurrence and interaction between toxin mixtures are shown in Figure 3 and Figure 4. Figure 3 shows that: (a) at minimum concentrations of FBs and DON (560.8 and 650.8 µg/kg, respectively), ZEN, regardless of the concentration, had no influence on broilers FCR, but, at maximum level of DON (2620.9 µg/kg), increasing ZEN concentration had an impact on FCR values; and (b) at maximum levels of FBs (3500.1 µg/kg) and minimum level of DON, even a slight increase in the level of ZEN drastically increased the value of FCR. However, the opposite effect was true when high level of DON was present. As shown in Figure 4, the presence of maximum level of DAS (899.8 µg/kg) and minimum levels of DON caused a sharp increase in FCR as the ZEN concentration increased. The assumptions for a valid linear regression in terms of linearity of variables, normality, multicollinearity and homoscedasticity were all met.

## 3. Discussion

For the first time, a longitudinal study was used to determine the extent of broiler chicken exposure to multiple mycotoxins and the impact on performance parameters. Other studies have evaluated this at a single point in time with only regulated mycotoxins and at levels that may not be applicable to all field conditions. Eighteen successive performance trials were carried out from May 2017 to December 2019. During the study period, four feed samples, comprising of starter, grower, finisher and withdrawal diets, were collected at the start of each performance trial and analysed for multiple mycotoxins using a validated QuEChERS-based LC-MS/MS method. No statistically significant difference was observed in the levels of mycotoxin detected in diet types, collected for each trial (*p* > 0.05). This is expected, as the same materials were used to formulate broiler diets (based on nutrient requirements) and were all stored under the same conditions. In total, 24 mycotoxins, produced by three fungal genera (*Fusarium, Aspergillus* and *Alternaria*), were detected in the analysed samples. Other researchers who carried out multi-mycotoxin surveys also found several mycotoxins co-occurring in feed samples [5,18,19,20]. In total, 524 finished feed samples collected worldwide were found to be contaminated with 235 metabolites including regulated and emerging mycotoxins [20]. Up to 57 mycotoxins were also found in 1113 finished feeds collected between 2012 and 2015 from 44 countries [18]. Out of the six EU-regulated mycotoxins, only DON, ZEN and FBs were found to contaminate feed samples analysed in the current study. FBs contaminated all the samples at an average concentration of 1.27 mg/kg, while DON and ZEN were found in 98% and 91% of the samples at mean levels of 1.38 and 0.11 mg/kg, respectively. All the analysed samples were compliant with EU guidance values for these toxins in poultry feed. In accordance with results obtained in this study, DON, ZEN and FBs were found to be the most prevalent mycotoxins in 8210 feed samples collected from Europe between 2008 and 2017 [19]. DON, ZEN and FBs contaminated 74%, 45% and 43% of samples, respectively, with approximately 1.5–20% of samples exceeding the EU guidance limits for mycotoxin in animal feed [19]. Outside Europe, Gruber-Dorninger et al. [29] reported a frequent contamination of 1041 finished feed samples from Africa with DON, ZEN and FB. Although levels of these toxins in most samples were below the EU guidance limits, aflatoxins (AFs) concentrations were far above EU maximum permitted limit [29]. In addition, DON (64%), FBs (48%) and ZEN (32%) were the most prevalent mycotoxins in samples from North America, while, in Asia, AFs, OTA, FBs, ZEN and DON were the most frequent mycotoxins in the feed samples [19].

With regard to non-regulated mycotoxins, an extensive review by Fraeyman et al. [13] showed that ENNs, BEV, APD, AUR, MON and EMD are the most frequently detected emerging mycotoxins in feed worldwide, while deoxynivalenol-3-glucoside, zearalenone-14-glucoside and zearalenone-16-glucoside are the common masked mycotoxins found in feed [30]. Deoxynivalenol-3-glucoside and zearalenone-14-sulphate were found to be the most prevalent mycotoxins in finished feed from Europe, at levels ranging from 0.09 to 0.25 mg/kg. In addition, MON, ENNs and BEV have been reported to be the most prevalent emerging mycotoxins in Europe at levels up to 2 mg/kg [18]. These data agree with results obtained in our study (Table 1). Regarding co-occurrence, approximately 90% of the samples were contaminated by more than 10 mycotoxins, and about 75% of the samples contained at least 20 mycotoxins. This is not surprising as finished feeds are blends of different materials from various sources. In addition, most of the mycotoxigenic fungi species can grow on feed materials simultaneously and are capable of producing more than one mycotoxin [7,31].

In terms of trial to trial variations, a distinct trend was observed in levels and pattern of mycotoxins found in feed samples analysed. Levels of DON, ZEN, BEV, ENNs, DAS and FBs were significantly higher in feed samples for performance Trials 1, 2, 8, 9, 14 and 15 (*p* < 0.05). No significant differences were observed in the levels of these toxins for other trials. Previous long-term monitoring studies also observed a large yearly variation in levels of mycotoxins detected in feed [19,32,33]. This suggests that factors including agricultural practices and climate variations may greatly influence the type of fungi that infect crops and mycotoxin production [7].

FCR also known as feed conversion efficiency, is a measure of the amount of feed required to produce one kilogramme of poultry meat (dressed carcass weight) [34]. Depending on the country and broiler breed, feed expenses account for approximately 60–80% of broiler chicken production costs and FCR is a pivotal indicator or benchmark for measuring broiler feed efficiency [35]. Birds with low FCR value are efficient feed user, while an increasing FCR value is generally considered an economic disadvantage. FCRs of birds in Trials 1, 2, 8, 9, 14 and 15 were significantly higher when compared with FCRs of birds in other trials (*p* < 0.05). Interestingly, high levels of DON (1.5–2.6 mg/kg), ZEN (0.18–0.24 mg/kg) and FBs (2.6–3.5 mg/kg) (albeit below EU guidance values), as well as high levels of DAS (0.08–0.9 mg/kg), ENNs (1.6–2.7 mg/kg) and BEV (0.03–0.5 mg/kg), were detected in feed samples given to birds in these trials. A strong positive correlation was observed between FCR of birds and exposure to DON (R^2^ = 0.85), FBs (R^2^ = 0.53), DAS (R^2^ = 0.86), ZEN (R^2^ = 0.92), ENNs (R^2^ = 0.60) and BEV (R^2^ = 0.73), suggesting mycotoxins may negatively impact birds FCR, as increase in levels of toxin mixtures resulted in higher FCR.

DON, ZEN, FBs, DAS, ENNs and BEV are all produced by *Fusarium* species, the most economically important mycotoxigenic fungi, prevalent in tropical, temperate and sub-tropical regions [19,21]. DON is the most abundant and important trichothecenes (type B), mainly produced by *Fusarium graminearum* and *F. culmorum*. The double bond between C-9 and C-10 as well as the 12,13-epoxide ring have been reported to be an important feature of trichothecene toxicity [36]. ZEN is a phyto-estrogenic compound with a phenolic resorcyclic acid lactone, also produced by *F. graminearum* and *F. culmorum.* ZEN can competitively bind to oestrogen receptors, leading to reproductive disorders [19]. FBs are produced mainly by *F. verticilloides* and *F. proliferatum* [19]. More than 28 FB homologues have been identified; however, FB1 is the most common and most characterised mycotoxin due to its toxicological relevance [37]. FB2 and FB3 are less prevalent and differ structurally from FB1 in the number and position of hydroxyl groups [19]. FBs can competitively inhibit the ceramide synthase, thus interfere with the biosynthesis of ceramides and sphingolipids of cell membranes, resulting in various adverse health effects in animal and human [38,39]. Only a few researchers have examined toxicity of DON, ZEN and FBs at levels equal to or below EU regulatory limits for mycotoxins in poultry feed (0.25, 5 and 20 mg/kg for ZEN, DON and FBs, respectively). Notwithstanding, the results reported to date are consistent with the results obtained in the current study. Broiler chicken exposed to 1–5 mg/kg DON experienced significant reduction in body weight and feed intake from Days 17 to 34 [39,40,41,42]. Similarly, Keçi et al. [43] reported a decreased body weight gain (up to 6%) in broilers fed DON-contaminated diet. Moreover, the meat weight of leg decreased linearly with increasing dietary DON concentrations (2.5–10 mg DON/kg feed) [43]. Regarding feeding behaviour and preference of contaminated diet, broilers exposed to 0.14 mg/kg (control), 2.27 mg/kg (low) and 5.84 mg/kg (high) of DON diets preferred the control DON diet to low and high DON-contaminated feed. In addition, birds on low and high DON diets spent more time at the feeder compared to control, with lower FCR (1.65) in control birds, than birds fed low (1.82) and high (1.94) DON diets [44].

The gastrointestinal tract is the first physiological barrier against food contaminants and the first target for toxicants such as mycotoxins. Consequently, intestinal toxicity of mycotoxins has attracted a lot of scientific interest [45]. Several researchers have reported an increased paracellular intestinal permeability and translocation of enteric microorganism (*Escherichia coli* and *Clostridium perfringen*) to the liver and spleen as well as alteration of corticosterone level, intestinal mucus layer and intestinal epithelial antioxidative mechanisms, when birds were exposed to DON and FBs at levels below EU guidance values [38,42,46,47]. Although broilers intestines were not investigated in this study, we suggest that the reduced feed efficiencies in Trials 1, 2, 8, 9, 14 and 15 may be due to deleterious effects of identified mycotoxins on intestinal mucosa structures and functions. A reduction in feed efficiency of chicken with an altered intestinal structure and function was recently reported in broilers fed fusarium-contaminated diet [43]. Furthermore, mycotoxins have been shown to induce gut satiety hormones including cholecystokinin and glucagon-like peptide-17–36 amide, resulting in anorectic response and reduced feed efficiency [48,49,50].

Compared to DON, ZEN and FBs, the other mycotoxins—BEV, ENNs and DAS—have not been regulated by EFSA. A scientific report on human and animal health risks related to the presence of BEV and ENNs in feed, by EFSA Panel on Contaminants in the Food Chain (CONTAM), showed that adverse health effects following poultry acute and chronic exposure to BEV and ENNs is very unlikely [12]. Moreover, few researchers who have investigated toxicity of these toxins in farm animals did not observe any deleterious effects, even at chronic exposures (up to 15 mg/kg for ENNs and BEV). Only Freyman et al. [51] reported a mild inhibition of enterocyte proliferation following broiler exposure to ENN B-contaminated diet (up to 3 mg/kg). Similar to BEV and ENNs, EFSA CONTAM Panel also assessed the risk of DAS; they considered it to be low for all animal species except chicken. They also expressed concern about co-occurrence and interaction of DAS with other fusarium mycotoxins [52].

Due to natural co-occurrence of fusarium mycotoxins, there have been an increasing concern about health effects of exposure to mycotoxin mixtures, because the toxicity of multiple mycotoxins cannot always be predicted based upon individual compound toxicity. Interaction of mycotoxins can lead to synergistic, additive and antagonistic effects [22]. In the current study, a multiple regression model was used to predict the interaction effect of mycotoxins DON, ZEN, FBs, DAS, ENNs and BEV on broilers feed efficiency. Due to the complexity of interpreting interactions with more than three continuous predictor variables, only three-mycotoxin mixtures or combinations were considered at a time, while the regression moderator variables were held constant at different combinations of minimum and maximum occurrence levels. Out of the ten possible combinations and interaction terms, only the interactions among mixtures of DON, ZEN and FBs as well as DON, ZEN and DAS had a statistically significant influence on broilers FCR. Figure 3 and Figure 4 show that ZEN levels ranging from 0.05 to 0.25 mg/kg are predicted to have no significant effect on FCR, when DON and either FBs or DAS are present at minimum levels, but occurrence of minimum and maximum levels of DON and FBs or DAS, respectively, adversely affected broilers FCR, when ZEN is present at concentration less than 0.1 mg/kg. Again, few studies have reported negative health effects in birds exposed to low doses of mycotoxin mixtures. Yunus et al. [53] fed broilers a diet contaminated with 1.68 mg of DON/kg and 0.15 mg of ZEN/kg for five weeks; they observed a reduced feed intake and body weight as well as an impairment of the intestine during the first three weeks of exposure. Moreover, the low DON–ZEN contaminated diet significantly modulated titres against Newcastle and infectious bronchitis viruses [53,54]. In addition, combination of DON (1.5 mg/kg) and FBs (<20 mg/kg) reduced dry matter and ilea energy digestibility of broiler chicken [39]. Exposure of birds to mixture of DON and FB1 at levels below EU guidance limits did not affect the performance of birds raised in healthy conditions. However, under conditions of pathogenic challenge, both mycotoxins altered the response of chickens to coccidiosis [55]. Conversely, chicken fed partially purified fungal extract containing DON, ZEN and FBs alone or combined, at levels equal to the EU guidance limits, did not show any significant changes in terms of performance and organ damage, when compared with birds fed uncontaminated diets [56].

Beside the toxicity of mycotoxins, they can also be referred to as “antinutrients”, due to their potential to interact with essential nutrients in the GIT [57,58]. A very low level of DON (<10 μmol/L) significantly inhibited the absorption of amino acids, sugars and lipids in epithelial intestinal cell line (HT-29-D4) [59]. In addition, broilers exposed to 2.5–5 mg DON/kg feed experienced a reduced dry matter intake, with a decline in calcium and phosphorus intake (up to 8%) [43]. Impaired absorption of nutrients has been strongly attributed to growth suppression in farm animals [40]. Importantly, fungi can reduce the quality, organoleptic properties and nutritional value, when they grow on food and feed [60].

With respect to mitigation of toxic effects of mycotoxins in poultry, several post-harvest strategies including the use of feed additives, have been employed [61]. Inorganic (clay minerals), organic (yeast cell wall and glucomannan) feed additives as well as microorganisms and enzymes are added to feed, with the sole aim of binding, inactivating or detoxifying mycotoxins, to reduce their bioavailability in farm animals [62]. Nevertheless, recently, Elliott et al. [63] showed that some of these substances, particularly clay minerals, may cause a wide range of negative health effects in farm animals, including an interaction with micronutrients and veterinary substances. Moreover, other researchers have reported inefficiencies of most commercial binders or detoxifiers, as well as their affinity for only a single mycotoxin [64,65].

## 4. Conclusions

Based on the authors’ knowledge, this is the first time a longitudinal study has been used to investigate performance-related effect of mycotoxins on broiler chickens, at levels representative of field conditions in many countries. The results obtained from this study show that prolonged exposure of broiler chickens to low doses of multiple mycotoxins below EU guidance limits can negatively influence broilers feed conversion efficiency. As it is well known that even small changes in feed efficiency will have a significant impact on profits, an effective intervention strategy to reduce levels of contaminants in feed and improve birds feed efficiency would help to reduce production costs and environmental emissions. In addition, our results indicate a need for continuous monitoring of mycotoxins in feed and further evaluation of health effects of low doses of mycotoxin mixtures, which may not actually induce any obvious clinical signs in farm animals but may predispose them to systemic disorders and reduce their performance.

## 5. Methods

### 5.1. Ethics and Animal Housing

The study was carried out in collaboration with a commercial poultry farm (Devenish Nutrition Limited), which operates in accordance with Northern Ireland animal welfare legislation governing poultry production. Since no biological samples were collected for research purpose or invasive procedures applied on the birds throughout the study period, ethical approval was not required for this experiment. Furthermore, national regulations permit this type of study to be undertaken without any ethical approval, provided no pain or suffering is induced during the trial. The study was conducted in a standard commercial poultry performance house in Northern Ireland, UK, between May 2017 and December 2019. The house is a deep litter system with a 48 ft × 200 ft Turkington wooden-frame house, split into 36 individual pens and set away from the walls to keep a uniform temperature throughout. The house temperature was regulated using a computer-controlled cooling–heating ventilation system and maintained at 25–32 °C. The lighting schedule was 24 h light during the first 36 h (24 h light:0 h dark), 18 h light:6 h dark at age 3–14 days, followed by a gradual increase in hours of light to reach 20 h light:4 h dark at age 21 days, which was maintained until slaughter age. Standard biosecurity measures including footwear change, foot dips and presence of sanitisers for workers and visitors were in place. Following clearance or end of a trial, the house was extensively cleaned and disinfested.

### 5.2. Data and Sample Collection

In total, 18 successive broiler performance trials or production cycles were carried out during the study period. For each trial, approximately 2200 individual of one-day-old Ross 308 broiler chicks were randomly distributed into 6 pens. Performance house conditions were relatively similar across trials to minimise bias. All the birds were given the same basal diet, which included a starter diet during Days 0–9, a grower diet during Days 10–20, a finisher diet during Days 21–29 and a withdrawal diet during Days 30–32. Basal diet, typically a wheat–soy-based diet, used throughout the trial was a standard in-house commercial formulation developed by Devenish Nutrition. Individual feed ingredients and other micronutrients were formulated to meet the maintenance and production requirements of birds. At the beginning of each performance trial, the feeding system was programmed to weigh and distribute each pen’s ration automatically, with feed and water given ad libitum. Pens also contained automatic scales that constantly record birds’ weight. All the chicks were weighed on arrival and performance parameters including live body weight, feed intake, mortality and FCR were recorded on a weekly basis. For mycotoxin analysis, up to 2000 g of each of the feed types (starter, grower, finisher and withdrawal) given to birds in each performance trial was collected (i.e., 4 feed samples per trial). For performance Trials 1–6, two starter feed samples were collected; thus, 78 poultry feed samples were collected during the study period. Feed samples were recorded, milled and stored at −20 °C prior to mycotoxin analysis.

### 5.3. Sample Preparation

The sample extraction procedure was based on a QuEChERS method with slight modifications [66]. Briefly, 1 g of homogenised sample was weighted into a 50-mL polypropylene tube, 5 mL of 1.5% formic acid in water (*v*/*v*) was added, and the sample was left to soak for 30 min. Then, 5 mL of acetonitrile were added, and the sample was vortexed for 30 min on multitube vortexer. Magnesium sulphate (2.5 g) and sodium chloride (0.2 g) were added, and the tube was immediately shaken for 30 s. After centrifugation at 3000× *g* for 5 min, to induce separation of the aqueous phase from the organic phase, 2 mL of the upper organic phase were collected and transferred to a 15-mL centrifuge tube containing 0.25 g of C18 silica and 0.5 g of magnesium sulphate. The tube was shaken immediately for 1 min and then centrifuged at 3000× *g* for 5 min. Subsequently, an aliquot of 1 mL of the sample was transferred into an Eppendorf tube and dried down under gentle stream of nitrogen. Residue was reconstituted in 1 mL methanol and filtered through a 0.2 μm PTFE syringe filter into a LC-MS/MS vial.

### 5.4. UHPLC-MS/MS Parameters

Chromatographic separation was performed using an Acquity UPLC I-class system (Waters, Milford, MA, USA). The analytical column employed was a 100 mm × 2.1 mm i.d., 1.6 μm CORTECS UPLC C18 column (Waters, Milford, MA, USA), with a flow rate of 0.4 mL/min. An aliquot of 2 μL sample extract was injected into the chromatographic system. Mobile phases consisted of water (A) and methanol/acetonitrile (B) (50:50, *v*/*v*), both contained 1 mM ammonium formate with 0.1% formic acid. The gradient elution program of the mobile phase was as follows: 0–3 min, 99% A/1% B; 3.1–6 min, 70% A/30% B; 6.1–7.5 min, 70% A/30% B; 7.6–9.0 min, 1% A/99% B; 9.1–11 min, 99% A/1% B; 11.1–13 min, 99% A/1% B. Column and sample temperatures were maintained at 45 and 15 °C, respectively.

The Aquity UPLC system was coupled to a triple quadrupole tandem mass spectrometer (Xevo TQ-S, Waters). Analysis of mycotoxins was carried out in multiple reaction mode. The mass spectrometry was operated with electrospray ion source in positive and negative mode. Cone voltage and collision energy were optimised by infusion of each individual analyte. The optimisation was performed using the automatic IntelliStart function. The capillary voltage was set at 2.5 kV; source temperature, 150 °C; desolvation temperature, 700 °C; desolvation gas flow, 1000 L/h; and cone gas flow, 150 L/h. For each mycotoxin, at least one precursor ion and two fragment ions were monitored, with the most abundant product ion selected for quantification and the second intense one for qualification.

### 5.5. Method Validation

Method validation was carried out by using a blank poultry feed sample. Extraction recovery and matrix-induced suppression/enhancement (SSE) were determined by spiking homogenised feed samples with three different levels (low, medium and high) of multi-mycotoxin standard solution. All samples were placed under fume hood overnight to allow evaporation of the solvent. Subsequently, the spiked and blank (control) feed samples were extracted as described above. A neat solvent was also spiked with the same levels of multi-mycotoxin solution. Recovery of each analyte was calculated as the peak area ratio of blank sample spiked before and after sample preparation multiplied by 100. SSE was determined by comparing the response of the matrix spiked with standards after extraction to a solvent standard, at the same concentration. SSE was calculated as the ratio of the peak area of the analyte in the matrix and solvent multiplied by 100.

For quantification, a six-point calibration curve was prepared by spiking blank matrix with appropriate amounts of multi-mycotoxin stock solution before and after extraction. The concentration range of the spiked samples were chosen to cover the respective limits of detection (LOD) of each toxin, estimated linear range of calibration, levels commonly found in naturally contaminated samples and legislation limits of regulated mycotoxins. Sensitivity was determined by LOD and limit of quantification (LOQ). LOD was defined as the concentration of each analyte that gave a peak with a signal-to-noise ratio (S/N) of 3, which was determined by injecting neat solvent standard solution at different concentration. LOQ was defined as the concentration of the analyte in spiked matrix giving a S/N ratio of 10. Precision was determined by intra-day precision (repeatability) and inter-day precision (reproducibility). Intra-day precision was carried out by analysis of three replicates on the same day at four different concentration levels, while inter-day precision was assessed by repeating the same procedure over five consecutive days and expressed as relative standard deviation (RSDr). The data were used to calculate within-laboratory accuracy and precision. The criteria for confirming a positive sample include: (a) a retention time within ±1.0% compared with the analyte in standard solution; (b) both qualifier and the quantifier with transitions above a S/N ratio of 10:1; and (c) the ion ratio of the quantifier and the qualifier transition within a defined target range set by Commission Decision 2002/657/EC [27].

### 5.6. Statistical Analysis

Multiple statistical analyses were performed using RStudio (R Version 3.6.3, PA). Correlation analyses between feed intake and body weight as well as feed efficiency and mycotoxins were performed using Spearman’s and Pearson’s correlation tests. Associations were considered statistically significant if the calculated *p*-value was <0.05. Multiple linear regression models with interaction were used to investigate the relationship between feed efficiency (dependent variable) and mycotoxins (independent variables). To reduce multicollinearity, all independent variables were mean-centred or standardised before analysis. Two-way analysis of variance was also applied, with significantly different means separated using Hsu’s multiple comparisons with the best and Tukey’s Honest significant difference test. Homoscedasticity of data was confirmed using Shapiro–Wilk test. The assumptions for a valid linear regression in terms of linearity of variables, normality and multicollinearity were also considered. R packages used for analyses include ggplot2, tidyverse, dplyr, tidyr, reshape, reshape2, car, corr, ggpubr, sjplot jtools and sjmisc. For data acquisition and processing of mycotoxin results, Masslynx and Targetlynx software 5.0 (Waters, Milford, MA, USA) packages were used.

## Figures and Tables

**Table 1:** Overview of occurrence and concentration (µg/kg) of mycotoxins in poultry feed samples collected between May 2017 and December 2019.

Mycotoxins	P/N (%) ^a^	Minimum	1st Quartile	Median	Mean	3rd Quartile	Maximum
Deoxynivalenol	77/78 (98)	650.8	790.6	898.5	1387.4	2359.4	2620.9
Zearalenone	71/78 (91)	50.3	53.7	78.4	116.1	201.5	240.9
Fumonisin B1	78/78 (100)	432.7	567.8	637.1	734.2	933.9	2300.2
Fumonisin B2	78/78 (100)	33.7	136.3	177.4	427.6	833.2	960.2
Fumonisin B3	78/78 (100)	32.9	50.5	66.5	115.5	173.8	320.1
Diacetoxyscripenol	60/78 (77)	99.2	136.2	148.0	361.7	750.3	899.3
Meleagrin	50/78 (64)	10.2	30.5	33.7	35.6	40.6	45.3
Aurofurasin	62/78 (79)	5.5	12.2	26.5	34.2	42.6	115.9
Tentoxin	60/78 (77)	11.9	28.9	32.1	30.6	33.4	59.1
Equisetin	55/78 (71)	5.7	9.3	10.2	10.2	10.9	13.5
Enniatin A	78/78 (100)	2.8	3.4	5.1	11.8	18.8	34.7
Enniatin A1	78/78 (100)	3.2	5.3	7.2	11.2	9.8	32.1
Enniatin B	78/78 (100)	180.3	200.2	329.2	800.5	1511.3	2190.2
Enniatin B1	78/78 (100)	25.8	41.4	64.5	139.9	224.4	396.0
Apicidin	66/78 (85)	<LOD ^b^	10.3	10.6	19.3	30.6	64.2
Deoxynivalenol-3-glucoside	45/78 (58)	12.3	16.9	33.8	46.5	49.2	145.7
Beuvericin	74/78 (95)	30.9	35.2	41.5	167.9	394.8	474.9
Roqufortine C	20/78 (26)	15.2	74.3	89.1	82.7	101.8	155.2
Alternariol	36/78 (46)	9.8	36.5	99.2	124.4	191.7	390.6
Citrinin	47/78 (60)	<LOQ ^c^	10.5	31.7	79.7	155.2	255.2
Emodin	65/78 (83)	<LOQ ^c^	5.7	9.8	10.2	10.9	30.3
Patulin	39/78 (50)	2.9	7.4	10.9	14.4	20.3	33.2
Moniliformin	60/78 (58)	10.3	22.0	24.7	30.5	44.6	62.5
3-Acetyl deoxynivalenol	40/78 (51)	<LOQ ^c^	13.5	35.6	42.1	68.4	90.1

^a^ Number of positive samples/total number of analysed samples multiplied by 100 (percentage of positive samples). ^b^ Below limit of Detection. ^c^ Below limit of Quantification.

**Table 2:** Average feed intake (FI) and body weight (BW) of broiler chickens across eighteen trials on Days 7, 14, 21 and 28, with Pearson’s correlation coefficient between body weight and feed intake.

Day	Avg. FI ^a^	Avg. BW ^b^	Correlation (r)
7	137.3	189.6	0.84 *
14	500.9	504.1	0.58 *
21	1153.5	994.6	0.45 *
28	2031.1	1525.2	0.04 **

^a^ Average feed intake (g). ^b^ Average body weight (g). * Statistically significant (*p* < 0.05). ** Not statistically significant (*p* > 0.05).

## Supplementary Materials

The following are available online at https://www.mdpi.com/2072-6651/12/7/433/s1, Figure S1: Percentage of poultry feed samples (collected between May 2017 and December 2019 samples) contaminated by regulated mycotoxins—Fumonisins (FB1 + FB2 + FB3) (100%), zearalenone (91%) and deoxynivalenol (98%), Table S1: Optimised MS/MS parameters for the analysed mycotoxins, Table S2: Performance characteristic of the LC-MS/MS method for mycotoxin in poultry feed, Table S3: Output of multiple regression model testing the main and interaction effects of mycotoxins (independent variable) on broilers feed efficiency (dependent variable).
