# Supplementary material for: Low Doses of Mycotoxin Mixtures below EU Regulatory Limits Can Negatively Affect the Performance of Broiler Chickens: A Longitudinal Study
Source: Toxins (Basel). 2020 Jul 1;12(7):433. doi: 10.3390/toxins12070433 (PMC7404967; doi:10.3390/toxins12070433)
Supplement: Supplementary file 1 [file toxins-12-00433-s001.pdf]

Supplementary Materials: Low Doses of Mycotoxin Mixtures below EU Regulatory Limits can Negatively Affect the Performance of Broiler Chickens: A Longitudinal Study

Oluwatobi Kolawole, Abigail Graham, Caroline Donaldson, Bronagh Owens, Wilfred A. Abia, Julie Meneely, Michael J. Alcorn, Lisa Connolly and Christopher T. Elliott

Chemicals

Magnesium sulphate (>99.5%), formic acid, Bondesil C18, Sodium chloride (>99.5%), ammonium hydroxide (≥25% in water), LC–MS grade methanol and acetonitrile were obtained from Sigma-Aldrich (Gillingham, UK). A Milli-Q system (Millipore, Molsheim, France) was used as a source of deionized water. Mycotoxins standards: Aflatoxin B1, Aflatoxin B2, Aflatoxin G1, Aflatoxin G2, Aflatoxin M1, Altenuene, Alternariol, Apicidin, Aurofusarin, Beauvericin, Citrinin, Curvularin, Deoxynivalenol, Enniatin A, Enniatin A1, Enniatin B, Enniatin B1, Equisetin, Fumonisin B1, Fumonisin B2, Fumonisin B3, HT2 toxin, Moniliformin, Nivalenol, Ochratoxin A, Ochratoxin B, Patulin, Penicillic acid, Skyrin, T2 toxin and Zearalenone were obtained from Romer lab, UK. Cyclopiazonic acid, Cytochalasin B, Fusarenon X, Fusaric acid, Gliotoxin, Roquefortine, Macrosporin, Meleagrin, Neosolaniol, Verrucarol, Verruculogen, Diacetoxyscripenol, Ergocornine, Ergocristine, Ergotamine, Sterigmatocystin, Stachybotrylactam, Tentoxin, Emodin were obtained from were obtained from Sigma - Aldrich (Gillingham, UK). Zearalenone-14-glucoside, Zearalenone-14-sulfate, 3-Acetyldeoxynivalenol, 3 and 15-Acetyldeoxynivalenol, Deoxynivalenol-3-glucoside were obtained from Endo science (Exeter, UK). From the solid standards, individual stock solutions were prepared at concentration of 1 μg/mL in appropriate solvent and stored based on manufacturer's instructions. For spiking experiments, a working multi-mycotoxin solution was prepared in methanol by mixing appropriate amounts of single stock solutions. This was stored in amber bottle at −20 °C and renewed every three months.

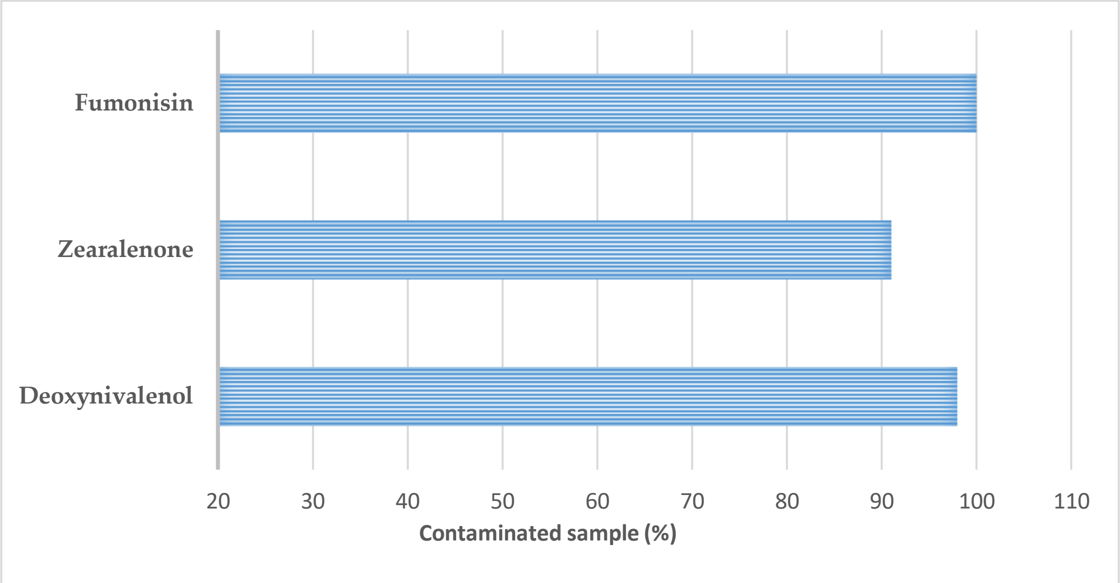

Figure S1. Percentage of poultry feed samples (collected between May 2017 and December 2019 samples) contaminated by regulated mycotoxins—Fumonisins (FB1 + FB2 + FB3) (100%), zearalenone (91%) and deoxynivalenol (98%).

Table S1. Optimized MS/MS parameters for the analysed mycotoxins.

Mycotoxins	Retention Time	Precursor Ion m/z	Cone (V)	Product Ion m/z[a]	Collision Energy (eV)[a]
Aflatoxin B1	5.09	313.3	44	241.1/285.0	36/20
Aflatoxin B2	4.76	315.1	68	259.1/287.1	30/24
Aflatoxin G1	4.61	329.1	14	200.0/243.1	36/26
Aflatoxin G2	4.35	333.1	12	189.1/245.0	36/28
Aflatoxin M1	4.22	329.0	40	229.1/273.1	42/22
Altenuene	4.64	293.2	8	257.0/275.0	12/8
Alternariol	5.42	259.1	64	127.9/185.1	40/28
Apicidin	7.86	624.3	26	112.1/211.2	56/40
Aurofusarin	7.12	571.1	4	484.8/556.1	34/24
Beauvericin	9.45	784.4	78	134.1/244.1	64/26
Citrinin	4.63	281.2	10	215.2/233.2	11/10
Curvularin	5.59	293.2	24	125.1/169.0	10/8
Cyclopiazonic acid	4.46	337.1	26	182.1/196.1	16/20
Cytochalasin B	6.22	480.3	42	91.1/444.3	54/14
Deoxynivalenol	2.65	297.2	30	231.1/249.1	10/8
Deoxynivalenol-3-glucoside	2.26	503.2	30	427.2/457.1	20/14
Diacetoxyscripenol	5.11	384.1	22	307.1/247.1	20/8
Emodin	5.78	271.1	74	115.1/197.0	42/26
Enniatin A	9.82	682.5	82	100.1/210.0	56/22
Enniatin A1	9.64	668.4	76	100.2/210.2	52/26
Enniatin B	9.18	640.4	76	86.1/196.2	60/28
Enniatin B1	9.45	654.5	76	86.1/196.2	58/24
Equisetin	9.18	374.2	28	140.0/170.0	40/22
Ergocornine	7.11	562.2	72	208.1/269.1	42/22
Ergocristine	7.46	610.2	36	208.0/223.1	48/36
Ergotamine	6.87	582.2	32	208.1/223.1	38/30
Fumonisin B1	5.70	722.4	74	334.4/704.4	40/28
Fumonisin B2	6.80	706.4	78	318.4/336.3	36/36
Fumonisin B3	6.31	706.4	8	318.3/354.3	36/32
Fusarenon X	2.89	355.1	14	175.1/229.1	24/16
Fusaric acid	3.16	180.1	10	65.0/162.0	30/10
Gliotoxin	4.84	327.1	2	111.1/263.1	24/8
HT2 toxin	5.78	425.3	10	215.2/245.0	15/10
Macrosporin	7.37	285.1	72	77.0/115.0	30/50
Meleagrin	4.62	434.2	24	261.2/403.2	38/14

[a]Values are given in the order quantifier/qualifier.

Table S1. Continued optimized MS/MS parameters for the analysed mycotoxins.

Mycotoxins	Retention Time	Precursor Ion m/z	Cone (V)	Product Ion m/z[a]	Collision Energy (eV)[a]
Moniliformin	3.42	97.0	10	97.1/41.3	4/10
Neosolaniol	3.18	400.2	22	185.1/215.1	20/14
Nivalenol	1.82	313.3	10	125.1/175.1	10/20
Ochratoxin A	6.93	404.1	36	102.1/227.1	66/34
Ochratoxin B	6.18	370.1	22	187.1/205.1	32/20
Patulin	1.65	154.9	30	109.0/127.0	8/6
Penicillic acid	2.90	171.0	20	111.7/125.0	14/12
Roquefortine C	5.37	390.2	54	193.1/322.2	24/18

Skyrin	8.61	539.2	8	469.0/521.1	32/24
Stachybotrylactam	7.36	386.2	66	150.1/178.1	42/34
Sterigmatocystin	6.96	327.1	2	111.1/263.1	24/8
T2 toxin	6.58	484.3	24	215.1/245.1	16/10
Tentoxin	5.91	415.2	28	199.2/302.2	12/12
Verrucarol	3.14	267.2	22	231.2/249.2	10/10
Verruculogen	7.46	494.3	26	199.2/352,1	38/12
Zearalenone	6.75	319.1	20	231.1/283.1	16/14
Zearalenonx10-14-glucoside	5.27	479.2	20	149.0/161.0	40/40
Zearalenonx10-14-sulfate	4.92	397.1	66	175.0/273.1	38/30
3-Acetyldeoxynivalenol	3.72	339.0	16	203.1/231.1	12/12
3 and 15-Acetyldeoxynivalenol	3.75	339.1	4	137.1/321.2	10/6

[a]Values are given in the order quantifier/qualifier.

Table S2. Performance characteristic of the LC-MS/MS method for mycotoxin in poultry feed.

**Mycotoxins**	**Recovery (%)**	**LOQ[a]**	**LOD[b]**	**SSE (%)**	**RSD_r (%)**	**Linearity (R^2)**
Aflatoxin B1	98	0.12	0.50	97	8	0.9992
Aflatoxin B2	101	0.12	0.25	98	6	0.9995
Aflatoxin G1	97	0.12	0.50	87	12	0.9987
Aflatoxin G2	96	0.25	0.50	102	10	0.9956
Aflatoxin M1	104	0.12	0.25	99	8	0.9990
Altenuene	98	5.00	10.00	89	7	0.9954
Alternariol	95	2.5	6.00	104	10	0.9947
Apicidin	89	5.00	10.00	97	12	0.9978
Aurofusarin	92	0.62	1.25	96	10	0.9976
Beauvericin	97	0.02	0.05	99	11	0.9982
Citrinin	10	5.00	10.00	90	6	0.9967
Curvularin	104	5.00	10.00	89	6	0.9945
Cyclopiazonic acid	97	1.25	2.50	87	13	0.9912
Cytochalasin B	94	1.25	2.50	82	8	0.9934
Deoxynivalenol	88	5.00	12.50	89	12	0.9967
Deoxynivalenol-3-glucoside	91	10.00	25.00	104	5	0.9948
Diacetoxyscripenol	97	5.00	10.00	99	7	0.9991
Emodin	91	1.25	2.50	106	7	0.9932
Enniatin A	105	0.02	0.05	92	4	0.9956
Enniatin A1	98	0.025	0.05	94	10	0.9977
Enniatin B	104	0.02	0.05	98	8	0.9992
Enniatin B1	102	0.02	0.05	91	6	0.9969
Equisetin	87	0.62	1.25	95	12	0.9989
Ergocornine	108	0.62	1.25	90	7	0.9956
Ergocristine	107	0.62	1.25	88	13	0.9947
Ergometrine	103	0.12	0.25	80	11	0.9951
Fumonisin B1	89	0.50	1.00	104	10	0.9934
Fumonisin B2	97	0.20	0.50	110	6	0.9972
Fumonisin B3	94	0.50	1.00	97	5	0.9994
Fusarenon X	96	25.00	50.00	90	11	0.9973
Fusaric acid	93	125.00	250.00	84	6	0.9965
Gliotoxin	90	5.00	10.00	89	5	0.9942
HT2 toxin	94	50.00	125.00	103	4	0.9922
Macrosporin	101	25.00	50.00	89	3	0.9961
Meleagrin	91	0.06	0.12	90	10	0.9929

[a]Limit of detection

[b]Limit of quantification.

Table S2. Continued—Performance characteristic of the LC-MS/MS method for mycotoxin in poultry feed.

Mycotoxins	Recovery (%)	LOQ[a]	LOD[b]	SSE (%)	RSD_r (%)	Linearity (R^2)
Moniliformin	104	5.00	10.00	91	6	0.9921
Neosolaniol	97	1.25	2.50	87	13	0.9962
Nivalenol	82	62.50	125.00	90	12	0.9973
Ochratoxin A	93	0.62	1.25	96	10	0.9964
Ochratoxin B	95	1.25	2.50	98	8	0.9944
Patulin	91	5.00	10.00	101	9	0.9921
Penicillic acid	91	5.00	10.00	76	13	0.9943
Roquefortine C	91	0.12	0.25	99	9	0.9952
Skyrin	105	1.25	2.50	90	8	0.9943
Stachybotrylactam	89	0.62	1.25	92	3	0.9996
Sterigmatocystin	93	1.25	2.50	80	5	0.9966
T2 toxin	97	2.50	5.00	93	8	0.9945
Tentoxin	104	1.25	2.50	90	10	0.9967
Verrucarol	109	0.02	0.06	94	10	0.9954
Verruculogen	107	0.25	0.62	104	14	0.9935
Zearalenone	92	2.50	5.00	75	4	0.9992
Zearalenonx10-14-glucoside	102	0.62	1.25	81	13	0.9921
Zearalenonx10-14-sulfate	109	0.02	0.06	79	10	0.9942
3-Acetyldeoxynivalenol	95	5.00	10.00	86	8.0	0.9931
3 and 15-Acetyldeoxynivalenol	95	12.50	25.00	107	8.1	0.9941

[a]Limit of detection

[b]Limit of quantification.

Table S3. Output of multiple regression model testing the main and interaction effects of mycotoxins (independent variable) on broilers feed efficiency (dependent variable).

Model	Predictors/Interaction Term	Estimate	SE	t	Pr (>|t|)	Adj. R^2	Overall *p*-value
1	Zearalenone	-2.786×10^{-03}	1.927×10^{-03}	−1.446	0.1788	0.84	0.01011
	Deoxynivalenol	-2.072×10^{-04}	1.335×10^{-04}	−1.553	0.1516		
	Fumonisins	-3.413×10^{-04}	594×10^{-04}	−1.316	0.2176		
	Zearalenone: Deoxynivalenol	2.206×10^{-06}	1.123×10^{-06}	1.965	0.0778		
	Zearalenone: Fumonisins	4.659×10^{-06}	1.701×10^{-06}	2.738	0.0109 *		
	Deoxynivalenol: Fumonisins	2.375×10^{-07}	1.235×10^{-07}	1.924	0.0233.		
	Zearalenone: Deoxynivalenol: Fumonisins	-2.437×10^{-09}	8.361×10^{-10}	−2.915	0.0124 *		
2	Zearalenone	7.728×10^{-04}	1.975×10^{-03}	0.391	0.7038	0.81	0.0004
	Deoxynivalenol	5.305×10^{-05}	1.177×10^{-04}	0.451	0.6616		
	Enniatins	-3.132×10^{-04}	2.045×10^{-04}	−1.532	0.1566		
	Zearalenone: Deoxynivalenol	-8.060×10^{-07}	1.488×10^{-06}	−0.542	0.5999		
	Zearalenone: Enniatins	2.991×10^{-06}	2.574×10^{-06}	1.162	0.2721		
	Deoxynivalenol: Enniatins	2.057×10^{-07}	1.087×10^{-07}	1.892	0.0877		
	Zearalenone: Deoxynivalenol: Enniatins	-1.315×10^{-09}	1.305×10^{-09}	−1.008	0.3374		
3	Zearalenone	6.258×10^{-04}	1.237×10^{-03}	0.506	0.624	0.87	6.56×10^{-05}
	Deoxynivalenol	3.078×10^{-05}	8.925×10^{-05}	0.345	0.737		
	Beauvericin	2.398×10^{-03}	2.573×10^{-03}	0.932	0.373		
	Zearalenone: Deoxynivalenol	6.972×10^{-08}	8.472×10^{-07}	0.082	0.936		
	Zearalenone: Beauvericin	5.916×10^{-06}	1.333×10^{-05}	0.444	0.667		
	Deoxynivalenol: Beauvericin	-4.942×10^{-07}	1.098×10^{-06}	−0.450	0.662		
	Zearalenone: Deoxynivalenol: Beauvericin	-4.455×10^{-09}	6.052×10^{-09}	−0.736	0.479		
4	Zearalenone	-1.550×10^{-03}	1.361×10^{-03}	−1.139	0.28141	0.91	0.001511
	Deoxynivalenol	-5.591×10^{-05}	8.594×10^{-05}	−0.651	0.53001		
	Diacetoxyscirpenol	-1.718×10^{-03}	5.914×10^{-04}	−2.905	0.21571		
	Zearalenone: Deoxynivalenol	6.868×10^{-07}	7.616×10^{-07}	0.902	0.38839		
	Zearalenone: Diacetoxyscirpenol	1.804×10^{-05}	4.441×10^{-06}	4.061	0.00128 **		
	Deoxynivalenol: Diacetoxyscirpenol	8.577×10^{-07}	3.115×10^{-07}	2.754	0.00535 *		
	Zearalenone: Deoxynivalenol: Diacetoxyscirpenol	-7.847×10^{-09}	2.162×10^{-09}	−3.629	0.00062 **		
5	Deoxynivalenol	-1.132×10^{-04}	1.749×10^{-04}	−0.647	0.531974	0.79	0.0008
	Fumonisins	-2.573×10^{-04}	3.456×10^{-04}	−0.745	0.473598		
	Enniatins	-4.052×10^{-04}	3.330×10^{-04}	−1.217	0.251571		

	Deoxynivalenol: Fumonisins	1.828×10^{-07}	2.022×10^{-07}	0.904	0.387052		
	Deoxynivalenol: Enniatins	2.462×10^{-07}	3.631×10^{-07}	1.173	0.267970		
	Fumonisins: Enniatins	5.353×10^{-07}	3.631×10^{-07}	1.474	0.171177		
	Deoxynivalenol: Fumonisins: Enniatins	-2.653×10^{-10}	1.986×10^{-10}	−1.336	0.211220		

SE = standard error; Adj. R2 = adjusted R squared.

Significant codes: 0 '***' 0.001 '**' 0.01 '*' 0.05 '.' 0.1 '.

Table S3. Continued—output of multiple regression model testing the main and interaction effects of mycotoxins (independent variable) on broilers feed efficiency (dependent variable).

Model	Predictors/Interaction Term	Estimate	SE	t	Pr (>|t|)	Adj. R^2	Overall *p*-value
6	Deoxynivalenol	1.060×10^{-04}	1.058×10^{-04}	1.003	0.339669	0.81	0.00038
	Fumonisins	8.747×10^{-05}	2.205×10^{-04}	0.397	0.699882		
	Beauvericin	3.026×10^{-03}	3.214×10^{-03}	0.941	0.368701		
	Deoxynivalenol: Fumonisins	-4.660×10^{-08}	1.009×10^{-07}	−0.462	0.654048		
	Deoxynivalenol: Beauvericin	-1.126×10^{-06}	1.357×10^{-06}	−0.830	0.426139		
	Fumonisins: Beauvericin	-7.177×10^{-08}	1.275×10^{-06}	−0.056	0.956234		
	Deoxynivalenol: Fumonisins: Beauvericin	4.341×10^{-11}	5.260×10^{-10}	0.083	0.935863		
7	Deoxynivalenol	5.003×10^{-05}	9.831×10^{-05}	0.509	0.6219	0.85	0.00014
	Fumonisins	-1.338×10^{-04}	2.071×10^{-04}	−0.646	0.5329		
	Diacetoxyscirpenol	-7.683×10^{-04}	5.262×10^{-04}	−1.460	0.1749		
	Deoxynivalenol: Fumonisins	7.932×10^{-09}	9.664×10^{-08}	0.082	0.9362		
	Deoxynivalenol: Diacetoxyscirpenol	1.478×10^{-07}	2.999×10^{-07}	0.493	0.6327		
	Fumonisins: Diacetoxyscirpenol	1.013×10^{-06}	3.545×10^{-07}	2.857	0.0771		
	Deoxynivalenol: Fumonisins: Diacetoxyscirpenol	-3.086×10^{-10}	1.673×10^{-10}	−1.844	0.0949		
8	Fumonisins	1.587×10^{-04}	2.290×10^{-04}	0.693	0.504054	0.75	0.0018
	Enniatins	9.895×10^{-05}	1.816×10^{-04}	0.545	0.597824		
	Beauvericin	2.801×10^{-03}	2.900×10^{-03}	0.966	0.357042		
	Fumonisins: Enniatins	-7.472×10^{-08}	1.459×10^{-07}	−0.512	0.619634		
	Fumonisins: Beauvericin	-1.360×10^{-06}	1.151×10^{-06}	−1.182	0.264572		
	Enniatins: Beauvericin	-1.350×10^{-06}	1.348×10^{-06}	−1.002	0.340165		
	Fumonisins: Enniatins: Beauvericin	6.820×10^{-10}	5.855×10^{-10}	1.165	0.271159		

9	Fumonisins	8.475×10^{-05}	1.892×10^{-04}	0.448	0.6638	0.83	0.00029
	Enniatins	1.305×10^{-04}	1.515×10^{-04}	0.861	0.4091		
	Diacetoxyscirpenol	1.610×10^{-04}	4.611×10^{-04}	0.349	0.7342		
	Fumonisins: Enniatins	-1.101×10^{-07}	1.092×10^{-07}	−1.008	0.3372		
	Fumonisins: Diacetoxyscirpenol	-3.078×10^{-07}	2.784×10^{-07}	−1.106	0.2948		
	Enniatins: Diacetoxyscirpenol	-5.169×10^{-07}	3.398×10^{-07}	−1.521	0.1592		
	Fumonisins: Enniatins: Diacetoxyscirpenol	3.551×10^{-10}	1.840×10^{-10}	1.930	0.0824		
10	Enniatins	-8.671×10^{-06}	1.089×10^{-04}	−0.080	0.938	0.73	0.002669
	Beauvericin	2.659×10^{-03}	2.911×10^{-03}	0.913	0.383		
	Diacetoxyscirpenol	-2.318×10^{-05}	5.160×10^{-04}	−0.045	0.965		
	Enniatins: Beauvericin	-9.502×10^{-07}	1.240×10^{-06}	−0.766	0.461		
	Enniatins: Diacetoxyscirpenol	1.401×10^{-07}	3.151×10^{-07}	0.445	0.666		
	Beauvericin: Diacetoxyscirpenol	-3.572×10^{-06}	3.883×10^{-06}	−0.920	0.379		
	Enniatins: Beauvericin: Diacetoxyscirpenol	1.294×10^{-09}	1.665×10^{-09}	0.777	0.455		

SE = standard error; Adj. R2 = adjusted R squared

Significant codes: 0 ‘***’ 0.001 ‘**’ 0.01 ‘*’ 0.05 ‘.’ 0.1 ‘’.
